# A Micro-Computed Tomography Technique to Study the Quality of Fibre Optics Embedded in Composite Materials

**DOI:** 10.3390/s150510852

**Published:** 2015-05-07

**Authors:** Gabriele Chiesura, Geert Luyckx, Eli Voet, Nicolas Lammens, Wim Van Paepegem, Joris Degrieck, Manuel Dierick, Luc Van Hoorebeke, Pieter Vanderniepen, Sanne Sulejmani, Camille Sonnenfeld, Thomas Geernaert, Francis Berghmans

**Affiliations:** 1 Department of Material Science and Engineering, Ghent University, Technologiepark 903, 9052 Gent-Zwijnaarde, Belgium; E-Mails: geert.luyckx@ugent.be (G.L.); Eli.Voet@UGent.be (E.V.); nicolas.lammens@ugent.be (N.L.); wim.vanpaepegem@ugent.be (W.V.P.); joris.degrieck@ugent.be (J.D.); 2 UGCT—Department of Physics and Astronomy, Ghent University, Proeftuinstraat 86, 9000 Ghent, Belgium; E-Mails: Manuel.Dierick@ugent.be (M.D.); Luc.VanHoorebeke@UGent.be (L.V.H.); Pieter.Vanderniepen@ugent.be (P.V.); 3 Brussels Photonics Team (B-PHOT), Vrije Universiteit Brussel, Pleinlaan 2, B-1050 Brussel, Belgium; E-Mails: ssulejma@b-phot.org (S.S.); csonnenf@b-phot.org (C.S.); tgeernae@b-phot.org (T.G.); fberghma@b-phot.org (F.B.)

**Keywords:** carbon fibre, defects, radiography, autoclave, prepreg

## Abstract

Quality of embedment of optical fibre sensors in carbon fibre-reinforced polymers plays an important role in the resultant properties of the composite, as well as for the correct monitoring of the structure. Therefore, availability of a tool able to check the optical fibre sensor-composite interaction becomes essential. High-resolution 3D X-ray Micro-Computed Tomography, or Micro-CT, is a relatively new non-destructive inspection technique which enables investigations of the internal structure of a sample without actually compromising its integrity. In this work the feasibility of inspecting the position, the orientation and, more generally, the quality of the embedment of an optical fibre sensor in a carbon fibre reinforced laminate at unit cell level have been proven.

## 1. Introduction

Amongst different sensing techniques, condition monitoring of composite structures using optical fibre sensors (OFS), and in particular fibre Bragg gratings (FBGs) appears to be the most suitable, because of their high accuracy (±1 µε), their immunity to electromagnetic interference and their small intrusive character when embedded in composite materials [1,2,3]. Furthermore, OFS technology has already proven to be useful as a monitoring tool for composite manufacturing [4]. Despite their small intrusive character, uncertainty still exists on the quality of embedding, on the exact position after production, on the embedded sensor’s measuring accuracy [3] and on their interaction with the composite during the whole life cycle of the structure [5,6,7].

**Figure 1:**
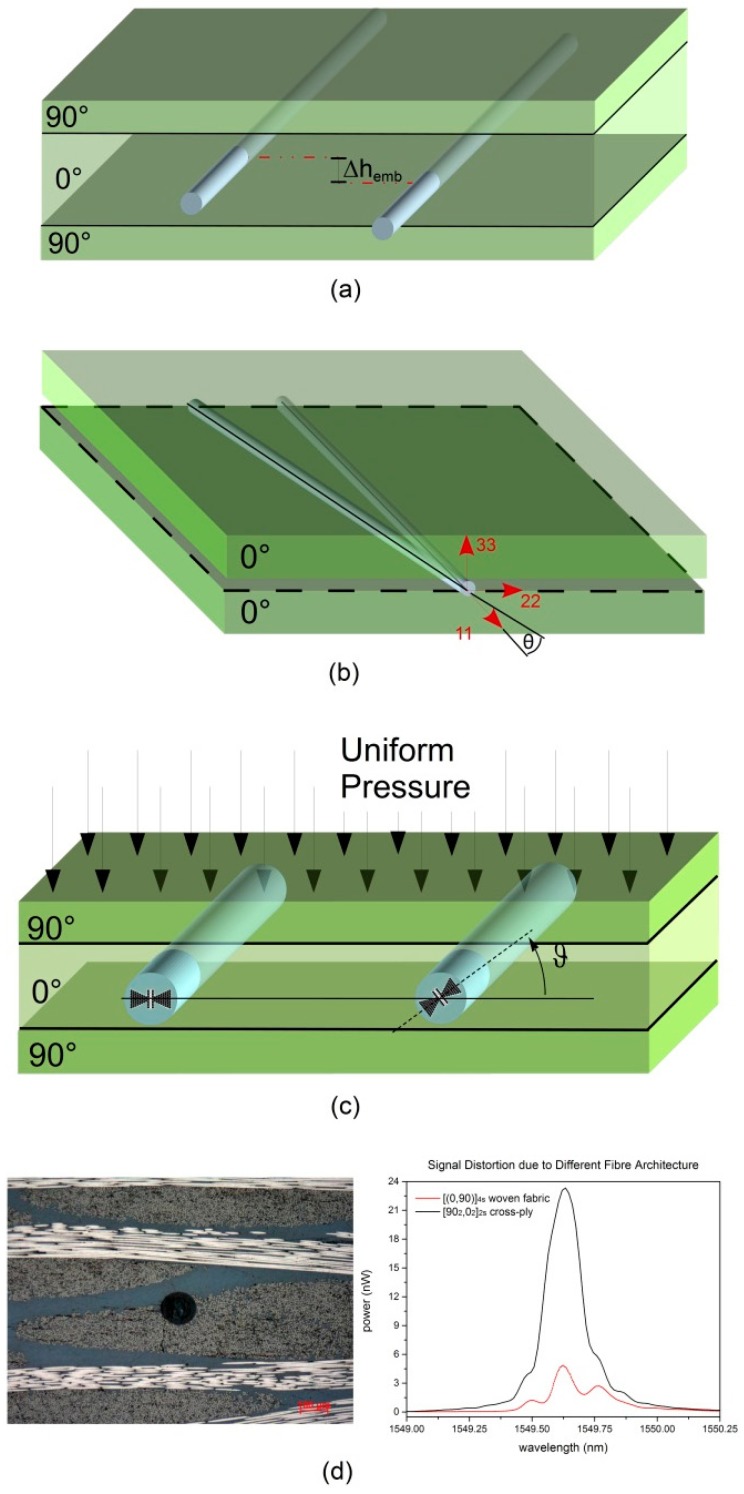
Schematic representation of (**a**) mal-positioning through thickness during embedding; (**b**) misalignment between sensor and reinforcing fibres direction on a UD layup; (**c**) orientation of a micro-structured MOF being controlled during laying down and (**d**) influences of the fibre architecture on the sensor readout: (left) polished cross section of a 125 µm fibre optic embedded in a 5-harness satin woven fabric Carbon-PPS laminate. (right) Peak distortion of the reflected signal for an FBG embedded in a Carbon PPS [(0,90)]_4s_ laminate and comparison with the reflected spectra of an FBG embedded in a cross-ply [90_2_,0_2_]_2s_ glass fibre reinforced laminate.

In Figure 1a schematic overview of the most important process parameters that need to be controlled carefully during the embedding is presented. First is the embedding depth of the sensor, which is an essential design parameter when evaluating a composite beam in flexion. The basic beam theory expresses the flexural stress component [8] at a given depth, as:

(1) $${\text{σ}}_{f,11}^{G}=\frac{{\text{M}}_{f,22}^{\text{G}}}{{\text{I}}_{22}^{G}}\;\times \;h_{emb}^{G}$$

where *M^G^_f,22_* is the bending moment acting on a beam at the grating location, *I^G^_22_* is the second moment of area of the beam’s cross-section at the grating location and *h^G^_emb_* is the embedding depth of the sensor, with respect to the neutral axis of the beam [8]. In fact, a malpositioning (Figure 1a) leads to an uncertainty on the measured strain. Secondly, in the case of a unidirectional (UD) laminate loaded along its longitudinal direction, a misalignment of the sensor with respect to the reinforcing fibres (Figure 1b) will lead to an underestimation of the real strain applied to the structure. Thirdly, a micro-structured optical fibre sensor (MOFs) designed to sense multi-axial strains is considered. A multitude of small air holes are created according to a dedicated design in the fibre cladding in order to improve its strain-sensitivities [9]. However, the orientation of such a sensor needs to be checked after embedding it in a composite laminate (Figure 1c) to avoid misinterpretation of its measured signal. As last, the composite layup and relative position of the sensor with the used fabric reinforcement materials affects the response of the sensor: Daggumati *et al.* have shown that an FBG embedded in a satin weave carbon/polyphenyl sulphide (PPS) composite responds both to non-uniform axial and transverse strains [10], causing distortion of the reflected spectrum (Figure 1d).

This leads to the need for a non-destructive inspection (NDI) after, or even during composite production, to assess the sensor integration or improve the embedding procedure. Several NDI techniques, such as thermography, ultrasounds, or radiography, are used in composites to detect and locate inclusions, defects and damage. Thermography, for instance, is mostly suited for detecting subsurface defects in composites; however distinguishing different types of damages remains difficult [11]. An ultrasonic C-scan is capable of locating damage inside composites [12], but the method does not allow detecting damages differently oriented with respect to the scanning plane (*i.e.*, defects of arbitrary geometry) [13]. In addition, it also results difficult to distinguish between defects and inclusion (*i.e.*, an embedded fibre optic). X-ray radiographic inspections instead, such as computed tomography (CT), are able to detect any defects or inclusion of smaller size in composite [14]. If strongly focused, this technique allows detecting cracks of a few hundreds of microns [15] and, therefore, is indicated as micro-computed tomography. The result of a scan is a 3D reconstruction of the inspected volume, which can be easily interpreted by anyone, without requiring any specific knowledge.

In this work a strongly focused micro-computed tomography, or micro-CT, was used to locate and visualize the quality of fibre optics embedded in composite materials. The aim of the study was to examine the potential of an in-house developed micro-computed tomography setup in order to address the points previously mentioned (Figure 1). As opposed to commercial CT scanners, this in-house developed piece-of-equipment presents the advantages of having high-resolution (up to 2 µm if strongly focused) and, at the same time, of being adaptable to any desired application [16]. On top of this, the in-house developed reconstruction software allows for better visualization and analysis of the results [17]. At the beginning, a description of the X-ray scanning facility is given, followed by four experimental cases. Firstly, the unknown position of an embedded fibre optic is determined with two radiographs. Secondly, the orientation of a MOF sensor is controlled after embedding. Thirdly, the embedding quality of a FBG sensor in a Carbon Fibre Reinforced Polymer (CFRP) cross-ply laminate is assessed and finally, the bending effect on a fibre optic embedded in a satin-woven CFRP thermoplastic material is described. For each of the cases the added value of this micro-CT technique is discussed.

## 2. Micro-CT Technique

High resolution X-ray computed tomography, or micro-CT, provides non-destructive 3D visualization and characterization, allowing one to investigate the internal structure of an object. It makes use of an X-ray beam which is sent through the object to measure the local linear attenuation coefficient for X-ray in the object, yielding morphological information. A common micro-CT setup consists of an X-ray source directing its rays towards the sample, which is usually mounted on a manipulator, and an X-ray detector positioned behind the sample. The transmitted X-rays hitting the detector yield a 2D projection or radiograph of the sample. A commonly used detector system is the so called “energy-dispersive scintillator detector”. Each pixel of the scintillator panel captures X-rays and converts them into light image. Fibre optics transfer this image to a charge-coupled device (CCD) sensor, where the light is transformed into an electric signal. The latter is further sent to an analogue-to-digital converter and stored on a hard-drive, as a radiograph. In order to recover the 3D distribution of the local attenuation within the sample, multiple radiographs need to be taken from different viewing angles, ideally covering 360°. After collecting all projection data, a reconstruction algorithm calculates cross-sections of the scanned object, which can be further rendered as a 3D volume using suitable visualisation software. This allows virtual inspection into the micro-structure. Two different implementations of micro-CT scanners are used in real applications: one where the sample is mounted on a rotational manipulator and scans are taken by rotating the sample between a stationary source and detector; in the other one the X-ray source and detector rotate around the stationary sample (e.g., medical applications).

**Figure 2:**
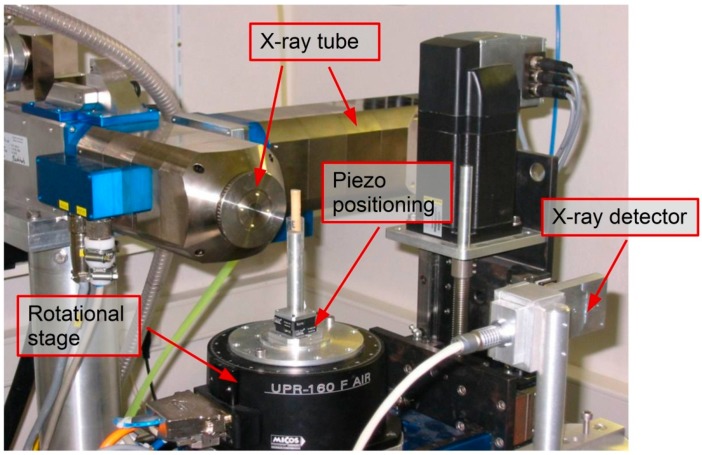
Micro-CT scanning facility from the “Centre for X-ray Tomography” of Ghent University (UGCT).

The main advantage of the second type of setup is the possibility to inspect a large object without the need of removing it from the main structure (*i.e.*, *in situ* checking), while the first is best suited for scanning small objects, using parameters and equipment which allow achieving higher resolutions (*i.e.*, micro-structural investigation). In the present work, the modular 900 nm CT scanner from UGCT—Centre for X-ray Tomography of Ghent University—having the first described configuration, was used [16]. An overview of the facility is given in Figure 2. The sample is fixed on a high precision piezo-positioning manipulator and rotates, allowing to obtain projections at different orientations. The X-ray tube is a FXE-160.50 dual head open type source from Feinfocus (Hamburg, Germany) with medium energy (up to 160 keV) and maximum power of 150 W. The detector chosen for the proposed applications is a 2520 V Paxscan a-Si flat panel (Varian, Palo Alto, CA, USA) with 1820 × 1460 pixels, 127 µm pixel size, covering a 20 × 25 cm^2^ area. The sample manipulator is an XYZ-theta CT system with ultra-precision air-bearing rotation motor (UPR-160F AIR, MICOS, Eschbach, Germany).

**Figure 3:**
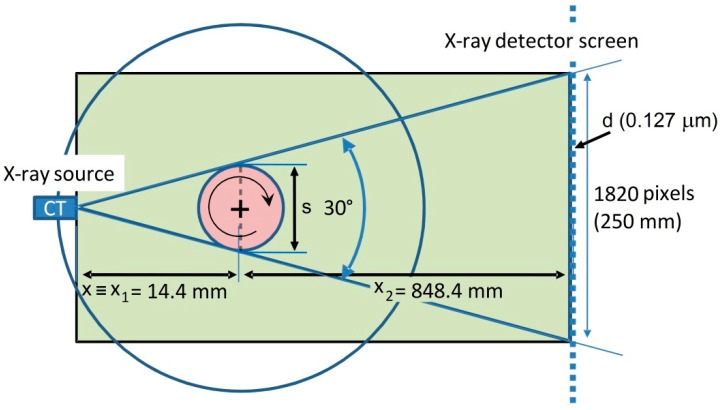
Schematic representation of the micro-CT setup. The distances defined in this work to achieve 4 µm spatial resolution between sample and X-ray tube and between sample and X-ray detector are shown.

The scanned samples, which measured approx. *w* × *t* × *l =* 10 × 2 × 250 mm in size, were positioned vertically on the manipulator and fixed at one end. Each of them had a fibre optic embedded in the symmetry plane along their length. The scanned volume was a small portion of about 3 × 2 × 4 mm^3^ in the central area of the sample. In order to achieve a good quality scan, several trials were performed and optimal parameters were selected. In Figure 3 a schematic representation of the chosen configuration is presented, with particular focus on the sample distances.

The resolution that can be achieved within the image is related to the magnification, which is defined as a function of the sample position *x* (*i.e.*, the distance between the X-ray source and the sample):

(2) $$M=\frac{x_{1}+x_{2}}{x}$$

where *x_1_* and *x_2_* the distances between the X-ray tube and the manipulator rotational axis and the distance between the manipulator axis and the detector, respectively. Thus, the resolution results from the following equation [18]:

(3) $$R=\frac{d}{M}+\left(1-\frac{1}{M}\right)s$$

*d* is the resolution (pixel size) of the detector and s is the focal spot size of the X-ray source [18]. From Equation (3), assuming that the detector resolution and spot size are fixed, one can see that the higher the magnification, *M*, one chooses, the better the resolution, *R*, will be. This implies that one can increase the resolution by placing the sample closer to the X-ray source, until the focal spot size becomes the limiting factor. On the other hand, one should also consider allowing enough room for the sample, which has to rotate freely without hitting the X-ray source. Based on the sample composition (*i.e.*, CFRP) and size of the scanned volume (*i.e.*, 2 × 4 × 3 mm^3^), the high voltage X-ray source was operated at 120 kV with 25 µA tube current for a resulting target power of 3 W and the focal spot size obtained was approx. 2 µm. Fixing the distance between the X-ray source and the specimen centre of rotation equal to x = 14.4 mm, the magnification becomes 59.917. The spatial resolution calculated with Equation (3) results of 4.0862 µm, while the voxel pitch obtained with these settings is of 2.1052 µm. The latter parameter does not consider noise effects or other artefacts, but it is often used to define the quality of a scan. In total 1500 projections were recorded over 360° and the data were reconstructed with the in-house developed software package Octopus [17]. 3D renderings were made with VGStudio Max.

A few more considerations need to be addressed for the reconstruction process, which becomes essential to remove artefacts typically involved in micro-CT. The attenuation of X-rays within the object is function of the material composition (*i.e.*, effective atomic number), density and thickness of the sample. The relation between the measured intensity of the transmitted X-rays (*i.e.*, grey scale value on the radiograph) and the attenuation coefficient µ is represented by the Beer’s law [19] as:

(4) $$\frac{\text{I}}{I_{0}}=e^{-\text{μ}s}$$

where *I* is the measured intensity, *I_0_* is the unattenuated intensity of the X-ray beam and *s* is the length of the X-ray path through the material [19]. This equation is only applicable for a single energy component beam through a homogenous material. In reality, the attenuation coefficient µ is also dependent on the X-ray beam energy: low energy X-ray photons are more easily attenuated than those with high energy. This can result in so-called beam-hardening artefacts, an overestimation of the attenuation in the outside areas of the sample. The reconstruction algorithm partly accounts for this artefact, but only works well if the boundary perimeter has a simple geometry [20,21,22]. In addition, small dense inclusions might also cause “star artefacts” due to scattering of low X-rays energy components [23]. The conical divergence of the X-ray beam projects an area on the detector screen instead of a line, thus reducing the definition of the scan. The reconstruction process itself is sufficient to compensate for the conical beam shape. However it has been noticed that reducing the step-rotation size (*i.e.*, number of projections in a 360° rotation) one can increase the signal-to-noise ratio by averaging a higher number of views. Moreover, imperfections or corrupted pixels on the detector screen can create darker rings on the reconstructed scans, known as “ring artefacts”. These can be removed by applying a ring filter during the reconstruction phase [24]. The scans presented in the following section have been reconstructed by applying a noise filter, a beam hardening correction, as well as a ring filter [25].

## 3. Results and Discussion

The proposed results always refer to CFRP samples. Different layups and OFS are discussed for each case study.

### 3.1. OFS Position Determination

**Figure 4:**
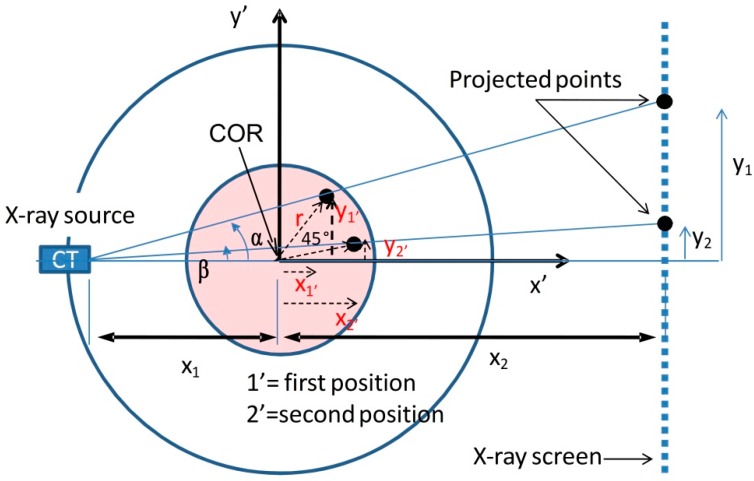
Schematic showing how to determine the position of a point located in the red circle by taking two projections at different orientations. The second one is taken after turning the red circle over 45°.

As already stated, keeping track of the sensor position after embedding is of relevance to define the real stress acting on the embedded fibre sensor and, accordingly, on the structure. Hereby the micro-CT is proposed as a tool to investigate this issue. As stated in the previous paragraph, the 2D cross-section of the scanned sample is the result of about 1500 radiographs taken at different orientations over 360°. This allows one to easily define the position of the embedded fibre, but normally the scanning time and the reconstruction process are time-consuming operations. Moreover, in some cases all this information is not needed and the only requirement is the fibre position. Theoretically, only two radiographs taken at different sample orientations are sufficient to define it. A generic approach allowing to define the position of a point in a scanned region will be considered at first, then this will be further extended to a real scan of a fibre embedded in a composite laminate. Figure 4 shows a schematic on how to geometrically determine the position of a point (*i.e.*, optical fibre) inside a red circle (*i.e.*, composite sample), which is rotated of 45° around its centre of rotation (COR). The projection of the point on the X-ray panel detector will shift when the red circle is rotated over this angle.

The y coordinates of the projections y_1_, y_2_ and the source-to-object and object-to-detector distances x_1_, x_2_ can be measured. Therefore the angles α and β can be derived as follows:

(5) $$\text{α}=arctg\;\left(\frac{y_{1}}{x_{1}+x_{2}}\right)$$

(6) $$\text{β}=arctg\left(\frac{y_{2}}{x_{1}+x_{2}}\right)$$

A system of five equations with the five unknowns *x*_1*'*_, *x*_2*'*_, *y*_1*'*_, *y*_2*'*_, r can be written:

(7) $$r^{2}=x_{1'}^{2}+y_{1'}^{2}=x_{2'}^{2}+y_{2'}^{2}$$

(8) $${\left(x_{2'}-x_{1'}\right)}^{2}+{\left(y_{2'}-y_{1'}\right)}^{2}=4\times {\left[r*cos\left(\frac{180{}^{\circ}-angle}{2}\right)\right]}^{2}$$

(9) $$y_{1'}=\left(x_{1}+x_{1'}\right)\times tg(\text{α})$$

(10) $$y_{2'}=\left(x_{1}+x_{2'}\right)\times tg(\text{β})$$

**Figure 5:**
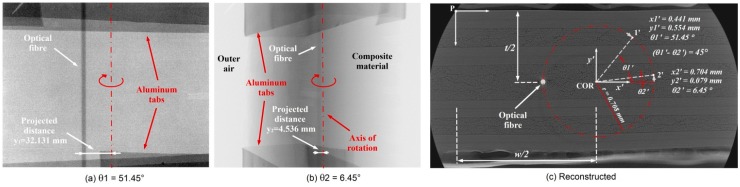
(**a**,**b**) Two X-ray projections taken at different angles (*i.e.*, −45° and 0°) clockwise with respect to the centre of rotation. The position of the OF is shifting from left to right due to its eccentricity with respect to the rotational axis of the manipulator. The darker regions above and below each projections are aluminium tape stripes attached to the sample; (**c**) Coordinates of the OF for the two different orientations were obtained using the formulas.

Solving the equations for a pair of orientations (*i.e.*, ϑ_1*'*_ and ϑ_2*'*_) will give a combination of solutions (under the form *x*_1*'*_, *y*_1*'*_, *x*_2*'*_, *y*_2*'*_), the direction of rotation allows then to discriminate the one of interest. Supposed that the centre of rotation of the sample is known (for easiness the sample could be fixed centred on the rotational axis of the manipulator), the embedding depth can be derived by knowing the overall sample dimensions. In Figure 5 two projections are taken at different orientations (*i.e.*, 51.45° and 6.45°) rotated of 45° in the clockwise direction with respect to the COR of the sample. Given the detector screen resolution of 0.127 mm and knowing that the detector screen is centred with the axis of the manipulator, the projected distance between the OF and the axis of the manipulator is of 32.131 mm for an orientation of 51.45°. This distance represents *y*_2_ and is needed along with *x*_1_ and *x*_2_ to determine the angle α. In the same way, for the second orientation of 6.45° the projected distance results of 4.536 mm and can be used to determine the angle β. Solving the system of equations (Equations (7)–(10)) will give two sets of solutions highlighting a pair of points which are positioned between the X-ray source and the rotational axis, and a pair of points positioned between the axis and the detector screen. Since the rotation of the sample was clockwise the chosen set of solutions is the second one, resulting in *r* = 0.708 mm, *x*_1*'*_ = 0.441 mm, *x*_2*'*_ = 0.554 mm, *y*_1*'*_ = 0.704 mm, *y*_2*'*_ = 0.079 mm as indicated in Figure 5c.

If the fibre embedding depth wants to be expressed with respect to a point on the outer surface of the specimen (for practical reasons in a real situation), as for example the point P on the upper-left corner indicated in Figure 5c, this can be achieved for any orientation ϑ given the radius *r*. After conveniently shifting the coordinates system towards point *P*, the coordinates of the COR can be expressed with respect to the sample width and thickness, as *x*_COR_ = w/2 and *y*_COR_ = t/2 and the fibre embedding coordinates will result as:

(11) $$x_{OF}=\frac{\text{w}}{2}+r\times \cos\left(\vartheta \right)$$

(12) $$y_{OF}=\frac{\text{t}}{2}+r\times \sin\left(\vartheta \right)$$

### 3.2. Micro-Structured OFS Orientation Control

For an axial stress state, the typical response of a single mode optical fibre sensor is a single reflected peak. The peak wavelength shift is sensitive to both applied strain and temperature. Moreover, a coated sensor is less sensitive to transverse strain if compared to an uncoated or stripped fibre (*i.e.*, the coating acts as a buffer), thus losing information on the real strain state of the sensor surroundings [1,26]. Depending on the application requirements, this can be seen as an advantage or a drawback.

**Figure 6:**
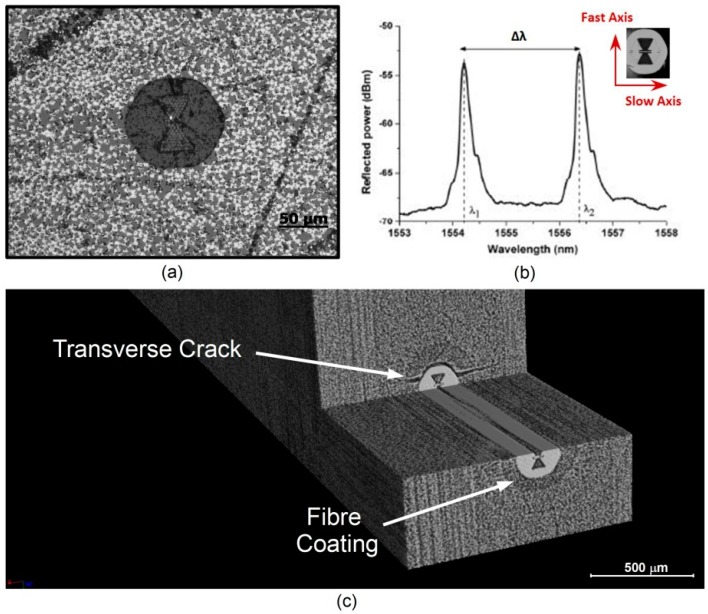
(**a**) Polished cross-section obtained by optical microscopy (0.8× to 20×) for a stripped micro-structured optical fibre (MOF) embedded in 8 UD layers of CFRP prepreg M18/M55J; (**b**) Typical wavelength response for a MOF, showing a fast and a slow peak; the shift is temperature independent; (**c**) 3D volume (*i.e.*, 3 × 2 × 4 mm^3^) reconstruction of a 2 µm voxel pitch micro-CT on an acrylate coated MOF embedded in CFRP prepreg M18/M55J cross-ply laminate. One can also notice a crack passing through the OF coating.

Micro-structured Optical Fibres (MOF) have a particular designed core structure filled by air holes, which gives them different sensitivities along their principal axes. Because of the air hole asymmetry in the design shown here (Figure 6a) modal birefringence is induced in the optical fibre, which results in two peak wavelengths being reflected by the sensor element. This particular fibre was specifically designed for peaks separation—which is directly linked to the fibre modal birefringence—to be sensitive to transverse strain, while insensitive to changes in temperature or axial strain [9,27].

In such case, the orientation of the sensor becomes relevant and needs to be controlled at the FBG location in order to avoid misinterpretation of signals. Luyckx *et al.* [28] have been investigating by means of a post-mortem cross sectional analysis the orientation and the consequent different strain response of several Panda fibres. In this section, the use of the micro-CT to get a feedback of the orientation for such a sensor is illustrated.

In Figure 6c, the results of 3D rendering of a micro-CT scan on a 125 µm MOF which has been embedded in a cross-ply CFRP prepreg M18/M55J laminate are presented. The fibre was placed between two 0° layers and the correct orientation was checked at its ends looking at the cross sections through a microscope. The purpose of the performed micro-CT was to investigate if the fibre presented a twist along its axis; by knowing the actual orientation, the correct calibration to axial and transverse load would be possible. The 3D rendering highlights a good orientation of the sensor along its axis and, even more important, a transverse crack evolving through the cladding/coating interface is highlighted. This emphasizes the importance of the technique for visualizing cracks or other defects otherwise hidden, as it will be further investigated in the next section.

### 3.3. Quality of the Embedding

**Figure 7:**
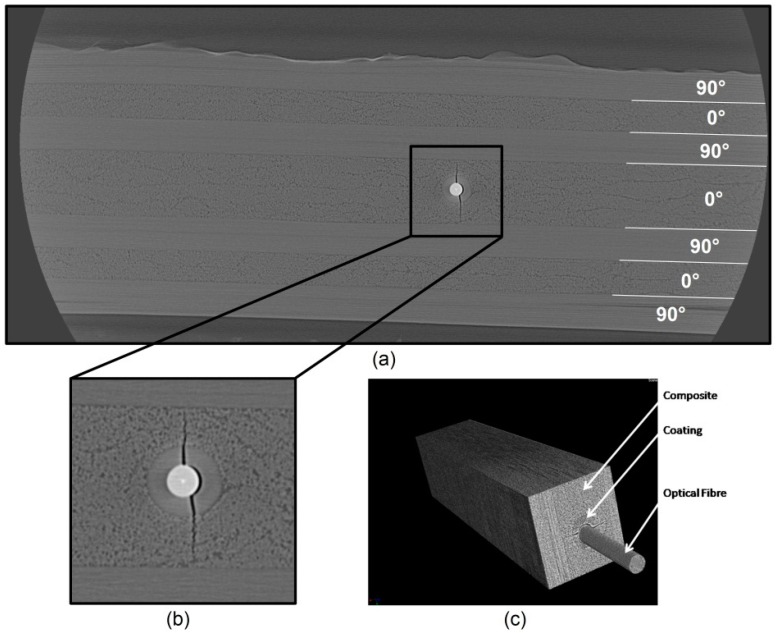
(**a**) 2D cross section reconstruction taken from a micro-CT performed on a CFRP prepreg M18/M55J cross-ply laminate with a coated OFS embedded in the mid-plane and (**b**) enlargement of its surroundings; (**c**) 3D volume rendering of approximately 1 × 1 × 4 mm^3^, which allows arbitrary virtual cross sectioning.

In this section, the results are shown for a micro-CT scan of the area around an optical fibre Bragg grating sensor, which has been embedded in a CFRP prepreg M18/M55J cross-ply laminate. The fibre provided (from FBGS Technologies GmbH (Jena, Germany) [29] in the framework of collaboration within the FP7 SmartFiber European project [30]) was a Draw Tower grating^®^ (DTG) sensor, coated with Ormocer^®^ (*i.e.*, Organig modified Ceramic) material and having a cladding diameter of 80 µm and a coating diameter of 190 µm. In Figure 7a a reconstructed and post-processed cross-section, referring to the above mentioned Micro-Computed Tomography, is presented along with its 3D rendering. The FBG sensor has been positioned in the laminate mid-plane, between two 0° layers (*i.e.*, cross-ply [90,0]_2s_). The laminate has been manufactured by an autoclave cycle imposing a maximum curing temperature of 180 °C, an external pressure of 5 bars and a vacuum level of about −85 kPa. After production, several samples were cut with a diamond saw from the plate at a proper width, according to the requirements of the micro-CT setup. Ideally in order to obtain a voxel pitch of 2 µm and a good quality scan, the material thickness surrounding the OF should be homogeneous in all directions, allowing the X-rays to travel across the same path (*i.e.*, same attenuation). On the other hand, technological limitations allowed us to reach a minimum cutting width of 10 mm. The resultant sample dimensions were of *w* × *t* × *l =* 10 × 2 × 250 mm. The final rendered volume was limited to about 3 × 2 × 4 mm^3^. The scan was repeated at different location on the sample, namely at the top, at the centre and at the bottom of its length. A reconstructed cross-section of the scan in the central region of the specimen is depicted in Figure 7a.

The quality of the 2D cross-section image is significantly improved by the noise reduction obtained by averaging a stack of 200 equally spaced slices (*i.e.*, 2 µm one from each other). The slices have been aligned and rotated with respect to the position of the OFS axis and an algorithm has been applied in order to improve the image contrast. In Figure 7b one can clearly note the coating around the fibre, as well as the central fibre core in whiter colour (8–10 µm in diameter). Moreover, around the optical fibre a clearly defined transverse crack is present (Figure 7c) and it appears to be propagating from the cladding-coating interface towards the composite. The same crack was present also in the results of the other two scans, meaning that it propagated along the whole fibre. Its initiation might be related to the build-up of residual strains during the cool down phase of the autoclave curing cycle. These transversal strains can be evaluated looking at the difference in the coefficients on thermal expansion (CTE) between the fibre coating material and the surrounding composite material. For this purpose a three-cylinder model with an infinite surrounding matrix [31] can be used and the residual stresses at the fibre/coating and coating/matrix interfaces can be derived as follows:

(13) $$s_{1}\;=\frac{1+s_{4}\left(1-2\nu_{c}\right)}{2s_{4}\left(1-\nu_{c}\right)}\frac{\left(s_{4}-1\right)\left(1+\nu_{m}\right)}{2s_{4}\left(1-{\nu_{c}}^{2}\right)}\frac{E_{c}}{E_{m}}$$

(14) $$s_{2}\;=\frac{\left(1+s_{4}-2\nu_{c}\right)\left(1+\nu_{c}\right)}{s_{4}-1}+\left(1+\nu_{f}\right)\left(1-2\nu_{f}\right)\frac{E_{c}}{E_{m}}$$

(15) $$s_{3}\;=\left(1+\nu_{c}\right){\text{α}}_{c}+\left(\nu_{f}-\nu_{c}\right){\text{α}}_{m}-\left(1+\nu_{f}\right){\text{α}}_{f}$$

(16) $$s_{4}\;={\left(1+\frac{t}{a}\right)}^{2}$$

(17) $${\text{σ}}_{int,m}=\frac{\left[\left(1+\nu_{c}\right)\left({\text{α}}_{m}-{\text{α}}_{c}\right)+s_{1}s_{3}\right]E_{c}\times ∆T}{s_{1}s_{2}-\frac{2\left(1-{\nu_{c}}^{2}\right)}{s_{4}-1}}=147.97MPa$$

(18) $${\text{σ}}_{int,c}=\frac{\left[\frac{s_{2}\left(s_{4}-1\right)\left({\text{α}}_{m}-{\text{α}}_{c}\right)}{2(1-\nu_{c})}+s_{3}\right]E_{c}\times ∆T}{s_{1}s_{2}s_{4}-\frac{2\left(1-{\nu_{c}}^{2}\right)}{s_{4}-1}}=-676.94\;MPa$$

where *E*, *ν* and α are the Young’s modulus, the Poisson ratio and the CTE, while the subscripts *c*, *m*, *f* refer to the coating (Ormocer^®^), the matrix (M18/M55J) and the fibre (silica), respectively. The terms *t* and *a* in Equation (16) are the fibre cladding radius and the coating thickness, respectively [31]. The mechanical and thermal properties of the three materials are taken from Table 1, Table 2, and Table 3 [32]. The CTE of the composite in the transverse direction has been derived from the classical laminate theory for the given layup and results of 0.686 × 10^−7^ K^−1^. The stresses arising at the interfaces are far above the limit of the M18/M55J transverse strength of 25 MPa (150 MPa in compression), thus justifying the initiation of the crack. However, this approach does not take into account the temperature dependency of the Young modulus or the change in volume induced by the resin curing and therefore it can only give a rough estimation of the interfacial stresses. One should always consider properly these aspects when embedding a fibre optic in a composite component, in order to limit as much as possible the residual strains. On the other hand, OFSs can also be of use to evaluate the residual stains in a composite laminate and thus lead to a better understanding of the several parameters influencing the phenomenon [33].

**Table 1:** Mechanical (compression) and thermal properties of M18/M55J prepreg.

E_1_	E_2_ = E_2_	ν_12_ = ν_13_	ν_23_	G_12_ = G_13_	G_23_	α_1_	α_2_
*[GPa]*	*[GPa]*			*[GPa]*	*[GPa]*	*[K^−1^]*	*[K^−1^]*
290	6.3	0.44	0.49	4.3	2.1	−1.02 × 10^−6^	35.2 × 10^−6^

**Table 2:** Mechanical and thermal properties of Ormocer^®^.

E	ν	α
*[GPa]*		*[K^−1^]*
1.44	0.32	142 × 10^−6^

**Table 3:** Mechanical and thermal properties of silica.

E	ν	α
*[GPa]*		*[K^−1^]*
72.4	0.16	0.55 × 10^−6^

### 3.4. OFS Bending in a Fabric Carbon-PPS

More than a position check, or a quality control, micro-CT represents a tool that helps to interpret strain profile at unit cell level [34,35]. The composite layup plays an important role related to the OFS embedding quality; as an example, a commercial 125 µm FBG has been embedded in a carbon polyphenyl sulphide (PPS) woven fabric—*i.e.*, Cetex [36]. Two different symmetric layups are compared, both using the same amount of “semi-preg” layers [29]. In the first case the OFS is aligned with the weft yarns of the layer in contact (*i.e.*, symmetry plane) and results in a [(0,90)]_4s_ layup. The second case on the contrary sees the OFS aligned with the warp yarns having a [(90,0)]_4s_ layup. Differences in compaction of the yarns during resin consolidation may lead to different strain profile. The obtained voxel pitch of the micro-CT scan was of 6 µm, and has been chosen as a compromise between obtaining a good quality image and, at the same time, being able to scan a complete unit cell volume (*i.e.*, unit cell length 7.4 mm). A total of four samples have been resized to these dimensions *w* × *t* × *l =* 10 × 2.5 × 250 mm and stacked side by side, so to be scanned all at the same time. The resultant scanned volume was of about 10 × 10 × 10 mm^3^.

**Figure 8:**
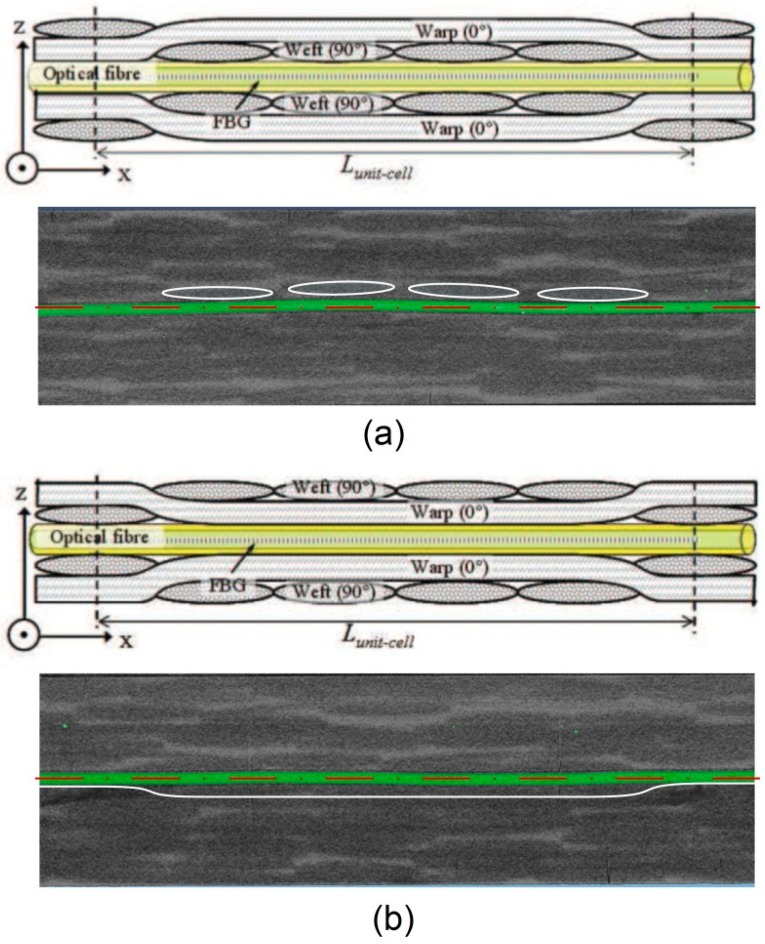
(**a**) Schematic and micro-CT side section of a 125 µm optical fibre embedded in a symmetrical stacked carbon-PPS [(0,90)]_4s_ layup; (**b**) Same schematic and micro-CT side section obtained for a [(90,0)]_4s_ layup. The Optical fibre has been highlighted in green colour. One can notice the bending of the fibre with respect to its axis (horizontal dash-dotted red line). The axis has been obtained from the envelope cylinder defined around the fibre. Finally, the white lines represent the envelopes of the bundle yarns of carbon fibre.

The difference for the two stacking sequences can be deduced from Figure 8, where a schematic shows a comparison between the layups considered, as well as a side section taken from the micro-CT rendering. For clarity, the fibre has been coloured in green; a pronounced bending along its axis can be noticed in both images (relative difference with the dashed horizontal line). This is a direct consequence of the fibre optic nesting around the weave fabric, which is compacted with 10 bar pressure and cured at 310 °C during the manufacturing of the laminate.

For each sample a spectrum of the embedded FBG was acquired with a commercial Bragg interrogator from FBGS (1 pm wavelength resolution). As suspected, the spectrum was distorted even though the samples had never been loaded. The sensor collects a mix of axial strain and different transverse components as a consequence of the yarns nesting. The bending effect on the FBG sensor is even more emphasized in the 3D micro-CT reconstruction around the grating location. The scan has helped to interpret the spectrum distortion (*i.e.*, lateral lobes and peak splitting), which built-up after curing of the resin.

**Figure 9:**
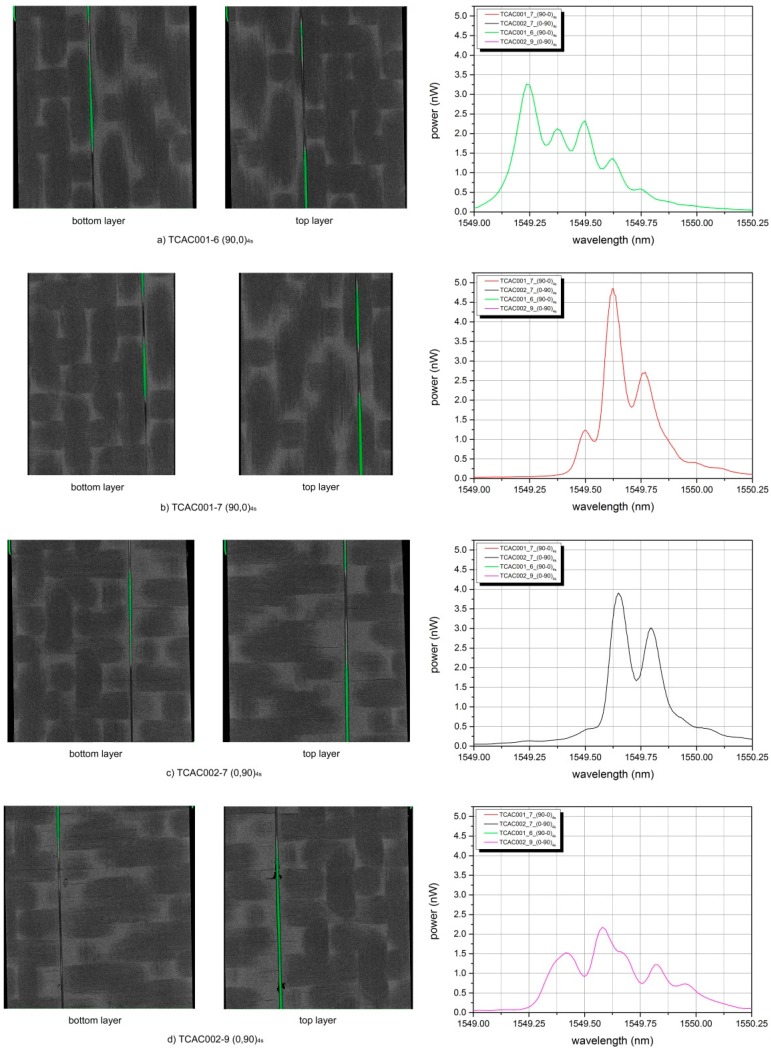
Sections along the stacking symmetry plane for four different carbon-PPS samples. Left: a section is taken from the 3D rendered volume of the reconstructed micro-CT along the woven-fabric layer just below the fibre optic; centre: the same section is taken just above the optical fibre on the upper woven-fabric layer; right: a spectrum of the optical fibre reflected signal is displayed. The first two cases (**a**,**b**) present the same stacking sequence [(90,0)]_4s_; while the last two cases (**c**,**d**) present the reversed situation [(0,90)]_4s_. Different embedding results are revealed, therefore different reflected spectrum have to be expected.

Further sections along the stacking plane at the OF location have been extracted from the 3D rendering, as shown in Figure 9. A total of four cases are presented: the first two (a,b) show the OFS embedded between two (90,0) layers (*i.e.*, the warp yarns are in contact with the grating), while (c,d) depict the OFS in between two (0,90) layers (*i.e.*, the weft yarns are in contact). On the left side one can find an image of a section relative to the lower half of the sample (layer immediately below OFS), in the centre the same image is taken for a section on its upper half (layer immediately above OFS) and on the right side the spectrum acquired from the embedded OFS is given. It can be noticed how the carbon-woven layers are not perfectly aligned one another, probably due to imprecisions during laying-up phase. In addition to this, it can again be noticed how the fibre optic bends due to the intrinsic carbon fibre architecture.

In case (a)-left the fibre results parallel to the warp yarn, but shifted to its edge. It can be noticed how the layer on top of it is shifted half a warp width to the left. The reflected spectrum results broadened and four different peaks can be identified. Case (b) presents the same layup: the section on the bottom layer shows the fibre at the edge of the warp yarn, while in the upper one the fibre is centred in the middle of the warp yarn. The resultant spectrum reveals again peak broadening with two well-distinct lateral peaks. Case (c) introduces the (0,90) layup: also in this case a shift between the warp yarns can be noticed from the lower and the upper layer. On the contrary, for the weft yarns the fibre seems positioned at the same location, namely on the yarn edge, both on the top and on the bottom layer. The spectrum exhibits again distortion resulting in a combination of peak splitting and shouldering, where the right peak exhibits lower intensity. Finally, the last case (d) presents a similar situation as per case (c), but a few relevant matrix cracks arise from the top-layer section. The reflected spectrum manifests lower power with respect to the other cases and there is a considerable peak broadening with three recognizable peaks. A clear distinction between the different cases cannot be made at this stage of the research, since the local nature of the fibre nesting depends on several factors (*i.e.*, fibre shift with respect to the warp yarn, matrix crack, layup, weft yarn position with respect to the FBG). However, what could be influencing more the response of the grating, and also the local strain profile of the unit cell, is the shift between two adjacent carbon woven plies. Normally during laying-down of the carbon woven plies this shift cannot be precisely controlled and therefore the response of the material could be different from case to case. In this sense, using data from fibre optics embedded in different samples which will then be tested could help validate a material model that accounts for this misalignment.

## 4. Conclusions

This work has presented a NDI technique, known as micro-CT, to investigate the quality of the embedding of an OFS in a CFRP laminate after production. This has set the basis for the further technique development to be applied after the production, as well as during operation, of a generic composite structure. The correct placement of the OFS during the embedding phase assures an accurate measurement (correct interpretation of the strain measured), reducing the possibility of having asymmetric stresses on the sensor. In this work, different optical fibre sensors in combination with different carbon fibre reinforced composite laminates layups have been considered. The micro-CT technique allowed to compare different embedding techniques and procedures, highlighting their benefits and drawbacks. For the MOF, the embedding quality and sensor orientation has been checked. Furthermore, it has been shown how transverse strains induced during manufacturing of a carbon fibre cross-ply laminate with an embedded fibre optic, may lead to initiations of cracks in the sensor vicinity. Damage assessment has not been considered in the current work, although the author intends to continue the research activities. In addition, it was also possible to investigate more complex fibre architecture of a woven fabric carbon-PPS laminate where a fibre optic has been embedded. This has revealed how the imprecisions during manufacturing (*i.e.*, yarn shift during plies stacking) can lead to a very difficult to interpret spectrum of the fibre optic sensor. However, addressing the local strain profile induced from resin crystallization during production for this complex fibre architecture is at this moment non-existent. The measured reflected spectrum seems to be affected by many variables, including embedding imprecisions and local fibre architecture. Furthermore, there is no evidence of a consistent difference in the spectrum profile for the two different stacking sequences considered.

In the frame of this research micro-CT has been applied successfully to CFRP hosting materials. For other materials, such as Glass Fibre Reinforced Polymers (GFRP), hybrid or bio-composite, the difference in the X-ray attenuation coefficient between fibre optic and hosting material might not lead to an appreciable contrast. It is an author intention to focus for the future work on micro-CT scans of GFRP and other materials. The overall goal of the present research is defining a reliable embedding method able to ensure adequate accuracy and repeatability that may be implemented according to industrial standards.
